# Multi-Faceted Analysis of Phase-Change Composite Intended for Autonomous Buildings

**DOI:** 10.3390/ma17112604

**Published:** 2024-05-28

**Authors:** Michał Musiał, Lech Lichołai

**Affiliations:** The Faculty of Civil and Environmental Engineering and Architecture, Rzeszow University of Technology, 35-959 Rzeszów, Poland; lech.licholai@prz.edu.pl

**Keywords:** autonomous building, heat storage, phase-change composite, molecular sieves, carbon recylate

## Abstract

This paper presents the long-term, holistic results of research into an innovative heat accumulator based on an organic phase-change material in the form of a mixture of aliphatic alkanes, molecular silica sieves, carbon recyclate and epoxy and cement matrices. The research included chemical testing of vacuum soaking of molecular silica sieves with a liquid phase-change material. The results proved an improvement in the heat storage efficiency of the heat accumulators due to the addition of carbon recyclate by 28%, while increasing the heat storage time by 134 min, and a reduction in PCM leakage due to the use of molecular silica sieves. In addition to its cognitive scientific value, another research objective of the work achieved was to obtain response functions in the form of approximating polynomials. They provide a useful, validated and verified tool to predict the physical and chemical characteristics of heat accumulators with different contents of individual components. As part of the ongoing research, technical problems related to leak-proofing assurance and matrix selection for organic phase-change materials were also solved. The solution presented is in line with the issues of efficient use of renewable energy, low-carbon and energy-efficient circular economy.

## 1. Introduction

The energy self-sufficiency of buildings while achieving zero carbon and the achievement of a circular economy are the leading goals of today’s construction engineering and related industries. Solutions to these problems are the subject of numerous scientific studies, which include active and passive solar systems and modern hybrid systems based on the extraction and storage of energy obtained from renewable sources. According to the analyses described in [1], the economic expenditure incurred for a residential building to achieve a passive building classification has twice the profitability in highly urbanised areas than in rural areas.

A modern approach to achieving building autonomy is to simultaneously reduce the demand for heat and cooling and cover this demand with energy from renewable sources. The first and intuitive way to improve the energy efficiency of a building, in addition to reducing heat loss, is to improve the control of the heating system, as explained in [2]. It is proven that the long-term savings from heat and cooling demand forecasting generate 13.4% and 10.7%, respectively, in the office and residential buildings analysed. These measures are to improve the thermal comfort of building occupants. This is extremely important because, according to [3,4,5], it is the instantaneous level of satisfaction with the thermal conditions of the building occupants that will determine the instantaneous (peak) heating and cooling demand. In turn, this has a direct impact on economic and environmental costs by increasing the carbon footprint of the demand. Storage of heat and cold by increasing the thermal capacity of building elements is one of the solutions used today to reduce the energy demand of buildings and improve their thermal comfort, as confirmed in [6,7]. Effectively increasing the thermal capacity of building components or their systems in an isothermal form and small footprint, according to [8,9,10], is possible using phase-change materials. Phase-change materials are incorporated both in the form of new composites applied conventionally or 3D printed [9] and for retrofitting existing buildings by injecting organic PCMs into walls and ceilings, as described in [11,12]. The use of phase-change materials in construction as building components or furnishing undoubtedly increases the thermal capacity of buildings and can contribute to improving indoor thermal comfort. Nevertheless, according to [13,14,15], whether the type of PCM used and the location of its application are chosen correctly in terms of realising the full potential of their phase transformation will determine the success of the whole project. No less important in this context is the provision of an efficient distribution system for the stored heat or cold to the indoor air.

Research evidence describing the beneficial effect of PCM on the thermal efficiency of buildings is presented in the following works. The example of the research described in [16] proves that the inclusion of a PCM with a capacity of 323 kJ in the forced ventilation systems of an airport concourse results in a reduction of the cooling heating time by 6 min and accounts for up to 48% of the heat distributed during this time. An example of the use of PCM in the form of inorganic sodium nitrate to increase a heat exchanger’s performance in industrial plants is presented in [17]. The phase change was 305 °C, the heat transfer was assisted by forced air flow, and the results confirmed an improvement in heat distribution of up to 30%. One example of the appropriate use of PCM is the PCM greenhouse wall system and the heat distribution system, which used heating microtubes, presented in [18]. The research study carried out for a reference solar day in an area of northern China proved an improvement of 95.35% in the heat capacity of the greenhouse wall and a 96.42% increase in the heat volume released after sunset. On the other hand, as presented in [19], the use of PCM in the form of ready-mixed micro-granulate for mortar or concrete mixtures is characterised by its ease of application and the absence of dangerous leakage but requires additional components to improve heat distribution by conduction. In addition, phase-change materials are also used to support low-temperature geothermal systems as high-density heat storage reservoirs. The research described in [20] shows that combining paraffin mixtures as PCMs in ground heat exchanger (GHE) systems results in a 77.8% improvement in heat storage efficiency when the thermal conductivity of the system is increased by 1 W/m and the melting point of the PCMs by 4 °C.

Attempts to solve the problem of insufficient thermal conductivity of PCM are presented in the following works. The increase in thermal conductivity, especially of organic phase-change materials, is one of the leading scientific issues related to their use in construction. In different contributions, conventional thermal conductors, such as copper or aluminium enclosures or foams [21,22], nano-mixes of carbon or graphite glass fibres, fullerenes, activated carbon [23,24], nanoparticles of transition metal oxides, e.g., titanium oxides TiO_2_, as described in [25], or using nano-liquid PCM systems, as described in [26,27], are used to enhance heat distribution within a solid PCM. An interesting example of increasing the efficiency of heat transfer within the PCM is the bionic system discussed in [28], based on the hierarchical arrangement of convective heat transfer channels between PCM granules. The results presented proved the achievement of a more uniform heat flow distribution than in the reference amorphous systems. A similar solution for cascading heat transfer between successive PCM centres was presented in [29]. A heat accumulator was made by 3D printing an aluminium matrix, and the heat-transporting working medium was a zeolite nanofluid. An example of a solution to both the problem of the low thermal conductivity of organic nonodecane and to ensure its tightness after melting is the conglomerate based on porous wood, activated carbon and organic PCM described in [23]. The solution is dedicated to heat storage capacity at approximately 84 °C, and the composite itself featured a heat capacity of 173.11 J/g with an increase in thermal conductivity relative to pure nonadecane by up to 3.81 times. In [25], a hybrid composite (GO-MgO-TiO_2_) that increased radiated heat transfer by 23% when in contact with PCM relative to pure PCM was presented. An interesting way of increasing heat transfer within the PCM is the use of hypergravity, as presented in [30]. Overloads ranging from 1 g to 9 g were applied to a PCM heat accumulator, which resulted in a fivefold increase in the liquid PCM flow velocity and, ultimately, a 60.24% reduction in its total melt time relative to the reference accumulator.

Examples of research work on the problem of producing tight composites with liquid PCM are presented below. Ensuring the constancy of a PCM’s application site regardless of its state of aggregation is another of the leading scientific problems associated with PCM use in construction. Regardless of the form in which the PCM is used, whether it is macro packets, capsules or micro-granulate in direct form, ensuring the integrity of the PCM will determine its suitability as a heat accumulator. Currently, polyurethane foams [31], metal sponges and foams [32], ceramic sinters [33,34], composite 3D prints [35], natural geological formations, such as carbides and silicates [36,37], and metallurgical waste [38] are used as porous matrices for composite phase-change materials. The work in [38] presents a study of a composite of paraffin and expanded clay as a matrix that provided both the required compressive strength and a porous medium capable of partially retaining PCM in its structure. In contrast, in [31], polyurethane foam was used as a porous matrix to hold the PCM, forming the cladding panels of a stud frame building. The results proved a 20% reduction in electricity consumption compared to conventional polyurethane panels. A solution with great potential for application is the use of non-Newtonian fluids, as described in [39], as a heat transfer support medium in porous media. In contrast, the authors of [35] investigated a PCM heat store, which was a 3D-printed mixture of ammonium graphite bicarbonate and paraffin. The resulting blend was a filament with a thermal conductivity of 19.27 W/m·K and good anti-leakage properties. Other solutions to increase the tightness of PCM composites are molecular sieves based on carbon [37,40] or silica [41,42,43]. The pore size ranged from a few to several hundred A, thus reducing the risk of PCM leakage, but at the same time, there was a risk of molecular sieves becoming brittle under the thermal stresses caused by the change in the aggregate state of the PCM. It is important to select the pore dimensions of the matrix for PCM, as pores larger than 0.5 mm allow for a significant increase in the amount of PCM applied, thereby reducing the strength of the composite, while smaller pores provide greater composite tightness. Among the composites of phase-change materials improved in terms of heat distribution and ensuring their tightness, those with satisfactory thermal insulation properties are also being investigated. The authors of [44] presented a composite of foamed concrete, exposed vermiculite and caprylic acid as PCM. The resulting composite was used as an infill for the external walls of a building and had a latent heat value of 69.16 J/g and a total thermal conductivity coefficient of 0.129 W/m·K. In contrast, the beaded composite of lauric acid as PCM and carbon fibres presented in [45] formed a network with a latent heat of 132.5 J/g, a thermal conductivity of 2.5 W/m·K, and a heat transfer efficiency of 15% higher than in an identical composite with a homogeneous structure. Another example of the use of a non-standard PCM geometry within a composite to improve heat transfer is presented in [46]. The seashell shape of the PCM used there allowed a lower perlite matrix content and a heat capacity of 136.40 J/g to be achieved. In contrast, the authors of [47] proposed a matrix of synthesis biocarbon obtained as a by-product of biomass processing as a matrix to increase the organic heat flow of PCM. Such a solution is more favourable from the point of view of life-cycle environmental and economic calculation, like LCA.

Articles describing possible solutions to the problem of freezing PMC composites are presented below. An important scientific problem and limitation of the use of PCM in temperate climates is the frost resistance of PCM composites, as highlighted in [48,49]. Using [50] as an example, it was noted that in composite mortars with a cement matrix and PCM in the form of encapsulated microgranulate, a microgranulate content of more than 30% resulted in a noticeable reduction in mechanical performance and a risk of failure of the frozen composite. An example of a solution to this problem could be the use described in [51]—of PCM granules embedded in a matrix of expanded graphite in cement matrix mortars. The presented research results proved that the danger of mortar failure due to frost is almost eliminated. Another solution to the problem of building partition freezing is presented in [52]. A mixture of directly applied organic PCM and a matrix of clayey was tested. Computational simulations proved an increase in the freezing time of an uninsulated external wall with clay and PCM by 4 h, compared to a conventional frame wall.

**Figure 1:**
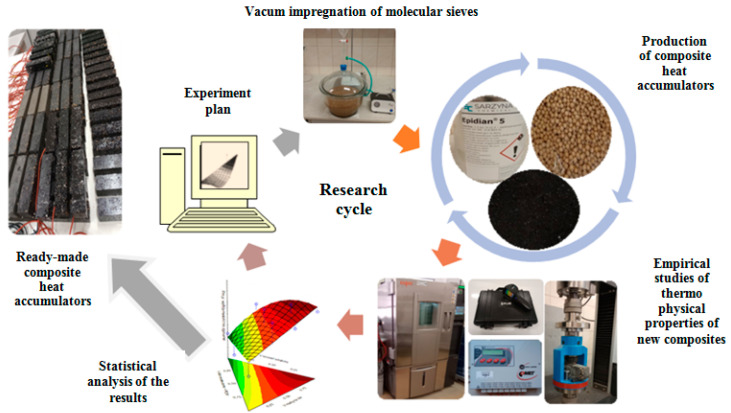
Graphic diagram of the various stages in the manufacture of PCM composites, molecular sieves, carbon recyclate and cement and epoxy matrices.

Summarising the review of the scientific literature, the authors see the need to solve the problem of easy and safe application of organic phase-change materials into the structure of buildings. The aim of the research will be to obtain new composites allowing for short-term heat storage thanks to PCM contained inside silica molecular sieves. The composites were verified for their thermal, strength and physical properties. The novelty of this work is to show the relationship between the proportions of PCM, matrix and carbon recyclate and their thermophysical properties, which has not been done in previous research works. A graphic diagram of the research cycle is presented in Figure 1.

## 2. Experimental Plan

To find an answer as to what effect the content of the individual components has on the efficiency of heat distribution and the strength of the newly designed composite, it was necessary to carry out an experimental plan. Because the individual plan input quantities (concentrations of the three mixture components) are dependent on each other, it was not possible to use incomplete experimental plans, such as the central or rotational compositional plan described in [53,54,55]. In this case, it was necessary to use a full, symplectic–centroid plan, which imposes limits on the values of the input parameters [56,57,58]. Plans of this type are used in numerous scientific papers, like [59,60,61], to determine the effect of each factor on the value of the response function.

For the above reasons, a full cubic experimental plan was made for the constrained mixtures before the experiments were carried out. Assumptions: three input quantities denoting the percentage composition of the three components and one output quantity denoting (heating time/compression strength).

### 2.1. Application of the Symplectic–Centroid Plan

**Figure 2:**
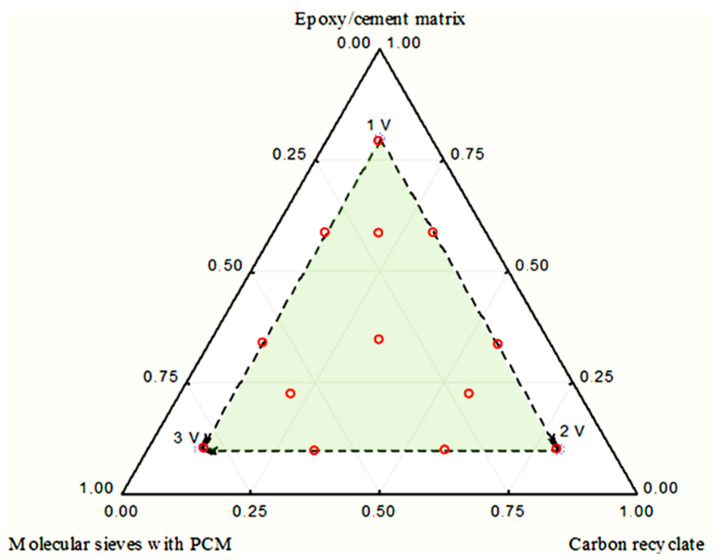
Gibbs triangle showing the range of variability of the input quantities of the full symplectic–centroid plan.

Its layout is organised by a full permutation of pure components, a permutation of binary mixtures and a permutation of ternary mixtures. In Figure 2, a Gibbs triangle is presented with the adopted concentration limits of the individual components of the composite heat accumulators.

A full cubic plan was chosen through a preliminary hierarchical analysis in the following way. The obtained results proved that the adopted full cubic plan, compared to linear, quadratic and special cubic, had the lowest pure error and regression coefficient. The form of the approximating function of the full cubic model is Equation (1).

(1) $$y=b_{1}\cdot x_{1}+b_{2}\cdot x_{2}+b_{3}\cdot x_{3}+b_{12}\cdot x_{1}\cdot x_{2}+b_{13}\cdot x_{1}\cdot x_{3}+b_{23}\cdot x_{2}\cdot x_{3}+\;d_{12}\cdot x_{1}\cdot x_{2}\left(x_{1}-x_{2}\right)+d_{13}\cdot x_{1}\cdot x_{3}\left(x_{1}-x_{3}\right)+\;d_{23}\cdot x_{2}\cdot x_{3}\left(x_{2}-x_{3}\right)+b_{123}\cdot x_{1}\cdot x_{2}\cdot x_{3}$$

with b_i_—directional coefficient ‘i’ of the input variable; x_i_—value of input variable ‘i’; d_ij_—directional coefficient of correlation of input variables ‘ij’.

The number of equations needed by the experimental plan to be solved included fourteen experiments. The requirement was to meet the condition of integrity of the multivariate regression run with a fixed component since the sum of the individual components in the mixture is a constant quantity.

### 2.2. Input Quantities and Ranges of Variation

To facilitate the experiments set out in the experimental plan, it was necessary to apply lower and upper limits that would result in each of the mixtures considered containing at least 10% and at most 80% of the individual components. The ranges of variation are shown in Table 1.

For the above assumptions to be considered into account in the experimental design and its subsequent analysis, it was necessary to apply upper and lower limits while maintaining the assumption that the sum of the percentage fractions of the individual components of the mixtures of interest must equal 100%. A summary of the experiments required to be completed is given in Table 2.

### 2.3. Output Quantities

The assumptions made for the experimental plan were to isolate a single input quantity. The completed experiments allowed verification of the influence of the individual input quantities (volume concentrations of the three components of the heat storage composites) on both the heat storage efficiency and the strength of the composites obtained.

Each input quantity was verified autonomously against the other but using a single experimental plan. The parametric result of the thermal efficiency index was determined by comparing the heating and cooling times of composite and reference specimens (containing the cement or epoxy matrix only).

(2) $$I_{H}=\frac{t_{C,0}-t_{PCM,0}}{t_{C,0}}$$

with t_c,0_—heating and cooling times of the reference sample; t_PCM,0_—heating time of the composite sample containing PCM.

**Figure 3:**
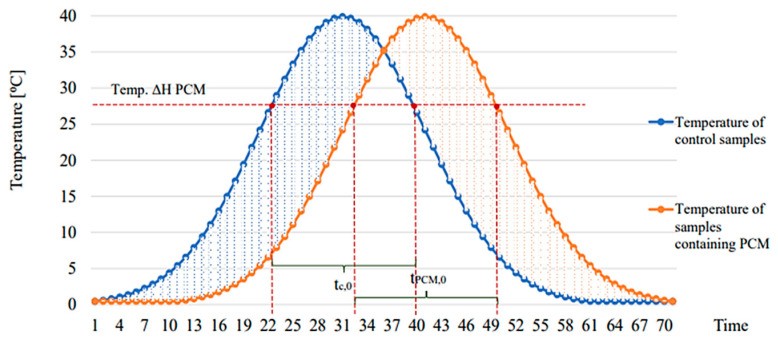
Diagram for the determination of the heating and cooling efficiencies of composite heat accumulators.

The heating and cooling times of the individual composite heat store specimens were determined by recording their temperatures during heat pulse loading tests, according to the diagram shown in Figure 3.

For the strength tests, on the other hand, empirically derived values of the characteristic bending strength were used as the starting quantity.

### 2.4. Verification of the Statistical Adequacy of the Adopted Model

To verify the statistical adequacy of the model adopted, a statistical test had to be performed against the empirical data. For this purpose, the Snedecor–Fischer test was performed, which involves comparing the population variance of given empirical results and the corresponding response function results. Fulfilment of the test consisted of checking whether the result of the null hypothesis of the test fell within the assumed 5% error area, according to Equations (3)–(5).

(3) $$H:\;\sigma_{a}^{2}⟺\sigma^{2}$$

(4) $$\sigma_{a}^{2}=\frac{1}{f_{1}}\sum_{u=1}^{n}{\left({\bar{x}}_{u}-{\hat{x}}_{u}\right)}^{2}$$

(5) $$\sigma^{2}=\frac{1}{f_{2}}\sum_{u=1}^{m}{\left({\bar{y}}_{u}-{\hat{y}}_{u}\right)}^{2}$$

where $\sigma_{a}^{2}$ is the variance of the inaccuracy of the actual measurements; σ^2^ is the variance of the inaccuracy of the calculated quantities.

Whereby verification of the adequacy of the model consists of calculating the test function, according to Equation (6), and comparing the result obtained with the maximum acceptable value of the critical variance, according to Equation (7).

(6) $$F=\frac{\sigma_{a}^{2}}{\sigma^{2}}$$

(7) $$F\leq F_{\alpha ,f_{1},f_{2}}$$

with f_1_ = (n − 1) degrees of freedom for $\sigma_{a}^{2}$; f_2_ = (m − 1) is the number of degrees of freedom for σ^2^; α = 0.05 level.

Additional verification of the result of the Snedecor–Fischer statistical test was then carried out using the quasi-Newton method, involving analysis of the scatter of the variances of the empirical quantities to the calculated ones. This is required because of the sensitivity of the statistical test used to peak measurements. The verification was carried out according to Equations (8) and (9).

(8) $$\min y=\sum_{t=1}^{n}{\left[\theta_{e}\left(t\right)-\theta_{k}\left(t\right)\right]}^{2}$$

(9) $$R^{2}=1-\frac{\sum_{t=1}^{n}{\left({\hat{y}}_{t}-\bar{y}\right)}^{2}}{\sum_{t=1}^{n}{\left(y_{t}-\bar{y}\right)}^{2}}$$

with y_t_—actual value of variable y at time t; ${\hat{y}}_{t}$—theoretical value of the explanatory variable; $\bar{y}$—arithmetic mean value of the explanatory variable.

**Figure 4:**
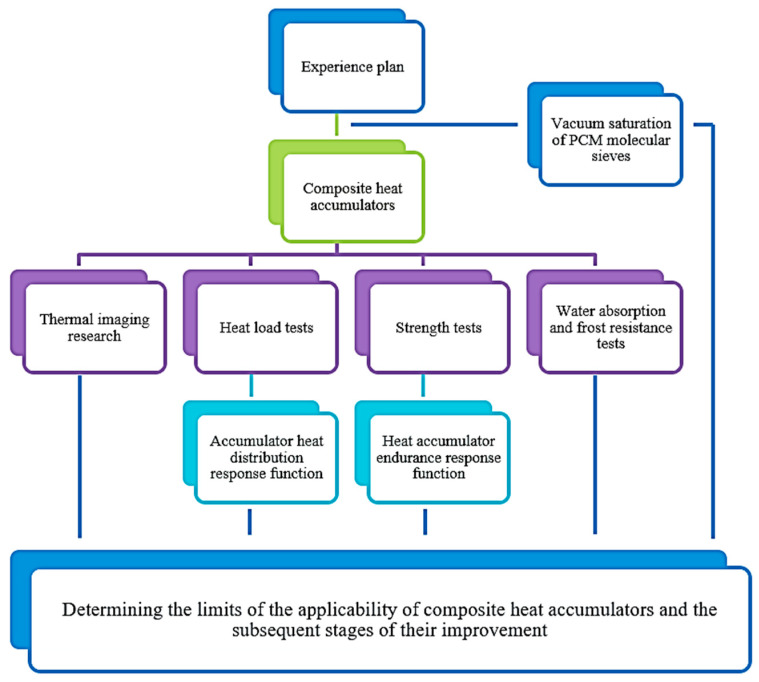
Graphic diagram of research and analysis.

The diagram of the empirical research performed and the statistical analysis of the results is presented in Figure 4.

## 3. Materials and Method

### 3.1. Materials

- Molecular silica sieves in granular form with a diameter of 4 mm and a pore diameter of 4 A, Alchem, Chempur, Poland, Toruń;
- Organic phase-change material in free form (mixing of aliphatic saturated hydrocarbons) RT28 HC, Rubiterm, Germany, Berlin;
- Epidian 5 epoxy resin with hardener Z1, Sarzyna Chemical, Poland, Nowa Sarzyna;
- Ready-mix concrete B25, Knauff, Germany, Perl;
- Coal recyclate, recovered from coke and metallurgical waste dump, Poland, Połaniec.

#### Research Apparatus

- Espec climate chamber, Japan, Osaka (accuracy of spatial temperature distribution ±1.0 °C);
- MS Data Logger MS6D, Comet, Czech Republic, Brno (measurement error ±1%);
- FLIR 7i thermal imaging camera, Canada, Vancouver (measurement error ±2%);
- Comtech testing machine, Taiwan, Tainan(measurement error ±2%);
- Heat flux density sensor, type FQA020C, Ahlborn, Germany, Holzkirchen (measurement error ±2%);
- PT1000 temperature sensor, Poland, Gliwice (measurement error ±0.3 °C).

### 3.2. Preparation of Test Samples

The ability of the developed composites to isothermally store heat was made possible by using a phase-change material. A limitation of its use in direct form is its low viscosity, which means that it can undergo uncontrolled leakage after melting. The solution to this problem was to soak a highly porous material, such as molecular silica sieves, in molten PCM. The high pore content of 5 A, combined with the cement–epoxy matrix, prevented the PCM from leaking after it was melted. To ensure a satisfactory level of saturation of the molecular silica sieves with the PCM, the entire process was operated at sub-atmospheric pressure.

**Figure 5:**
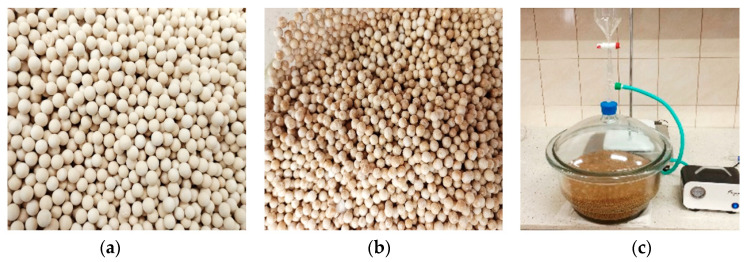
(**a**)—molecular silica sieves before PCM saturation; (**b**)—molecular silica sieves after PCM saturation in vacuum; (**c**)—laboratory bench during the vacuum saturation of molecular sieves with liquid organic PCM.

The soaking process consisted of placing 7.72 kg of molecular silica sieves in a 10 dm^3^ vacuum desiccator and placing 7.72 kg of molecular silica sieves required to make 96 heat accumulator samples. PCM RT28HC, previously melted and heated to 40 °C, was then added in an amount sufficient to immerse all the molecular sieves contained in the desiccator. A vacuum pump was connected to the closed and sealed desiccator, which started to reduce the internal pressure. At the same time, the desiccator and its contents were heated so that the PCM did not solidify. The pressure inside the desiccator was reduced by 90 kPa, triggering the release of air bubbles. The entire process was operated for 3 h until the formation of new air bubbles ceased. After the process was completed, the molecular sieve pellets soaked in PCM were drained of excess liquid PCM and cooled to 20 °C. Photographic evidence of the laboratory bench and molecular sieves before and after their saturation with PCM is presented in Figure 5.

#### Preparation of Samples with Cement and Epoxy Matrices

The adopted experimental plan required a minimum of 14 experiments to be carried out so that the response function could be determined. For the heat load, frost resistance and bending strength tests, 48 specimens (3 × 14 specimens plus additional 6 reference specimens) with a cement matrix and a further 48 specimens with an epoxy matrix were made. The heat accumulator test samples were standard rectangular standard bars measuring 40 mm × 40 mm × 160 mm. The individual samples were made according to the standard procedure described in [62], while the content of the individual components (PCM-saturated molecular sieves, carbon recyclate, cement or epoxy matrix) was determined in the experimental plan presented in Figure 2. The epoxy matrix was made from a 10:1 weight ratio combination of Epidian 6 and hardener Z1.

**Figure 6:**
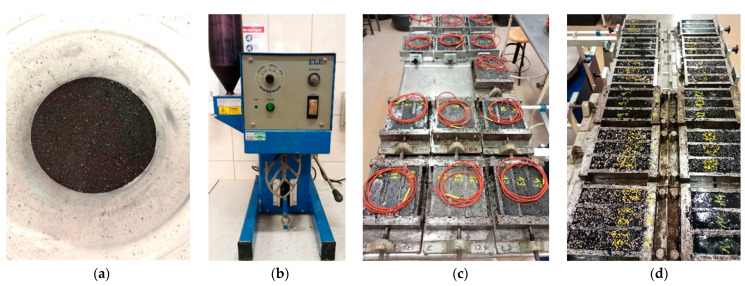
(**a**)—coal recyclate; (**b**)—concrete mix power mixer; (**c**)—cement matrix heat accumulator samples; (**d**)—epoxy matrix heat accumulator samples.

The bars with heat accumulators were made in standard steel moulds, and the individual components of the composites were combined in a mechanical mixer. To be able to determine the thermal dynamics of the specimens, a PT1000 temperature sensor was placed inside each of the 14 types of specimens with the cement and epoxy matrices. Photographs of the production of the test samples are included in Figure 6.

The samples were unmoulded 24 h after fabrication, following which the samples were weighed and cured. The epoxy matrix samples were stored at 20 °C and 60% humidity for 14 days, while the cement matrix samples were cured by immersion in deionised water for 28 days at 20 °C. Following the above steps, the heat accumulator samples were subjected to heat load, frost resistance and destructive tests.

### 3.3. Description of Heat Load Tests

**Figure 7:**
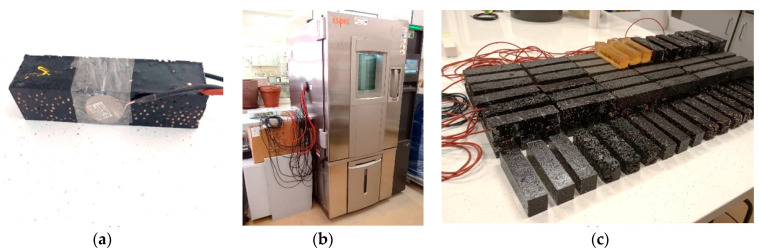
(**a**)—heat accumulator test sample with sensors connected; (**b**)—Espec climate chamber and Comet recorders, shown while testing; (**c**)—photograph of the heat accumulator samples produced.

Heat load tests were carried out in an Espec climate chamber with temperature control. The prepared samples were additionally fitted with a PT1000 sensor and an FQ A0206 heat flux density sensor. The sensors of each of the test samples were connected to two 16-channel COMET MS6D recorders, and measurements were recorded every 2 min. The heat loading conditions of the specimens inside the climate chamber were established as four repeated heating and cooling cycles, during which both cement matrix and epoxy matrix specimens were tested under identical conditions. Each test cycle consisted of a five-hour stabilisation of the temperature of the samples to 19 °C, followed by heating to 35 °C over a period of two hours, and a further five-hour cooling of the samples to 19 °C. The entire test cycle lasted 12 h, during which time the air temperature change inside the climate chamber was recorded. Photos of the sample with applied temperature and heat flux density sensors, the climatic chamber during the tests and all laboratory samples taken are shown in Figure 7.

The yellow samples in the drawing are reference samples made of pure epoxy resin to compare their thermophysical properties with the obtained composites.

### 3.4. Description of Qualitative Examination with a Thermal Imaging Camera

**Figure 8:**
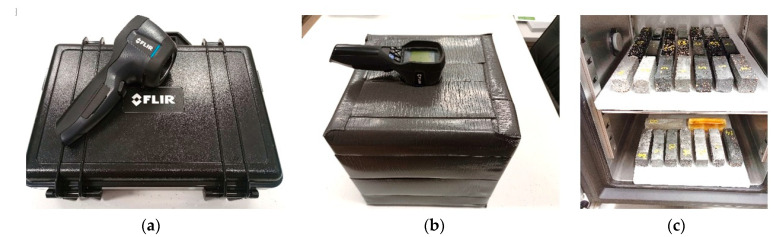
(**a**)—FLIR 7i thermal imaging camera; (**b**)—thermal imaging test benches; (**c**)—heated heat accumulator samples.

For a qualitative comparative analysis of the charging and discharging of accumulator heat, tests were carried out using a thermal imaging camera. Testing took place using a cubic enclosure made of 5 cm thick extruded polystyrene. As a result, thermal images of the samples were taken in an unlit space with negligible airflow. Measurements were carried out at an emissivity setting of ɛ = 0.95. The measurements consisted of six thermal images of each of the cement and epoxy matrix heat accumulator samples tested. Initially, all samples were heated in the climate chamber to 35 °C, and then their thermal images were taken after 3, 6, 9, 12 and 15 min at a fixed internal air temperature of 20 °C. Photographs of the test stand and apparatus are included in Figure 8.

### 3.5. Description of Frost Resistance Tests

**Figure 9:**
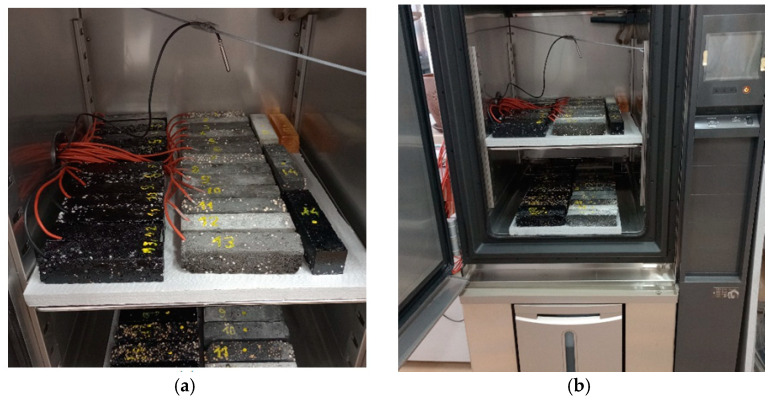
(**a**)—connected composite heat accumulators prepared for frost resistance testing; (**b**)—climate chamber with test samples of heat accumulators.

The next step in the research into the PCM heat accumulators was to evaluate the change in their thermal, physical and strength properties under repeated cooling and heating. The tests were carried out in an Espec climate chamber by running 28 cooling and heating cycles for all the heat accumulator types tested (28 sample types, i.e., 14 with cement matrix ones and 14 with epoxy matrix ones). This was a separate pool of test samples of composite heat accumulators, which allowed subsequent comparative analysis of identical numbers and types of reference samples. Each of the 28 temperature cycles inside the climate chamber took 12 h and included a four-hour stabilisation of the temperature of the samples to 20 °C, followed by a two-hour cooling period from +20 °C to −10 °C, a four-hour holding period of holding at −10 °C, followed by a two-hour period of heating from −10 °C to +20 °C. During the frost resistance testing of the specimens, both the specimens with embedded temperature sensors and the specimens used later for destructive testing were subjected to the experiment. Changes in temperature values inside the heat accumulator samples were acquired using two Comet MS6D recorders with a 2-min sensor output logging interval. Photographs of the frost resistance test performance on composite heat accumulators containing PCM, molecular sieves and carbon recyclate are presented in Figure 9.

### 3.6. Description of Absorbability Tests

**Figure 10:**
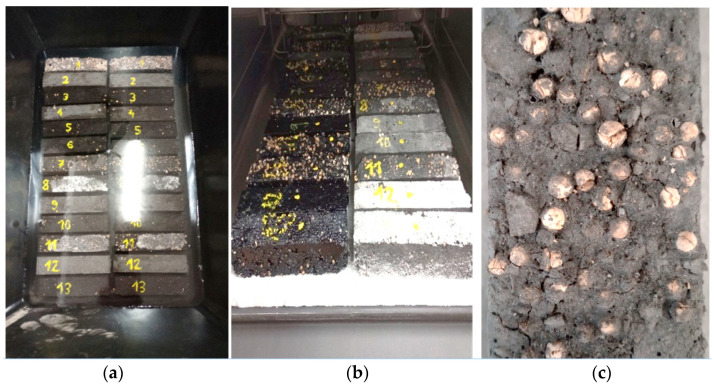
(**a**)—soaking of heat accumulator samples; (**b**)—temperature stabilisation of samples in the climate chamber; (**c**)—cracks in molecular silica sieves found in some samples.

Complementary tests were carried out to verify the absorbability of each of the heat accumulator types considered, both cement and epoxy matrix ones. The testing consisted of determining the difference in weight of the individual samples before and after a 28-day period of soaking in deionised water. The temperature of the water surrounding the samples was maintained at 20 °C. During the tests, the samples were analysed for the emergence of failure in the molecular silica sieves due to prolonged exposure to water. Photographs of the heat accumulators during the absorbability tests are included in Figure 10.

### 3.7. Description of Destructive Testing

**Figure 11:**
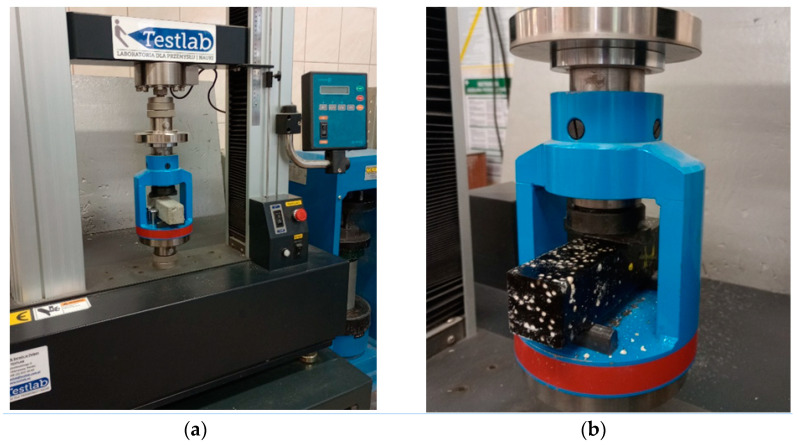
(**a**)—destructive testing of a cement matrix heat accumulator; (**b**)—destructive testing of an epoxy matrix heat accumulator.

The destructive tests included testing the bending strength using the standard method in [62] for three-point bending on a Testlab testing machine. The test specimens were divided into four distinct groups and subjected to bending strength tests. These were reference cement matrix specimens, cement matrix specimens subjected to cyclic freezing and thawing, reference epoxy matrix specimens and epoxy matrix specimens subjected to cyclic freezing and thawing. During the tests, the value of the critical force of failure and the limiting stress value determined by the apparatus were recorded. The tests were performed at an air temperature of 21 °C with a variation of ±2 °C. The results of the test formed the basis for the determination of part of the planned response functions, as part of the executed experimental plan. A photograph of the destructive testing is included in Figure 11.

### 3.8. Description of Anomalies Observed and Associated Phenomena

During the production of heat accumulators and testing, anomalies were found in the PCM-saturated molecular sieves. The anomalies consisted of the formation of defragmentation cracks in individual molecular sieves, resulting in the simultaneous release of the PCM contained therein.

**Figure 12:**
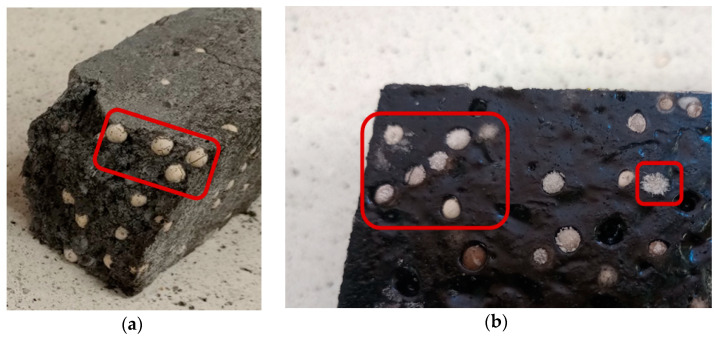
Found examples of anomalous molecular sieves saturated with PCM; (**a**)—cracking of molecular sieves; (**b**)—leakage of PCM from non-matrix encased molecular sieves.

Molecular sieve cracking was found among samples containing a significant ratio of cement matrix (above 50% of the sample volume), as well as in samples with the epoxy matrix in the case of incomplete matrix coating of silica sieve granules. Failure of the silica sieve granules in the samples with a high cement matrix content was already observed during the matrix setting. It should also be noted that the samples with a high cement matrix content were characterised by a significant temperature increase to values of 45–55 °C. At the same time, samples with the epoxy matrix increased their temperature to 35–40 °C during polymer setting, which did not damage the molecular sieves in the composite structure. Therefore, the aqueous environment was the destructive factor affecting the durability of the molecular silica sieves saturated with organic hydrophobic PCM. Photographs of the anomalies and defragmentation of PCM-saturated molecular sieves found are shown in Figure 12a,b.

## 4. Results

### 4.1. Results of Empirical Studies

#### 4.1.1. Heat Load Tests

During the fabrication of heat accumulator samples based on PCM-saturated molecular sieves, their temperature changes were recorded. The temperature was measured in the central part of the cross-section of each heat accumulator made according to the experimental plan.

**Figure 13:**
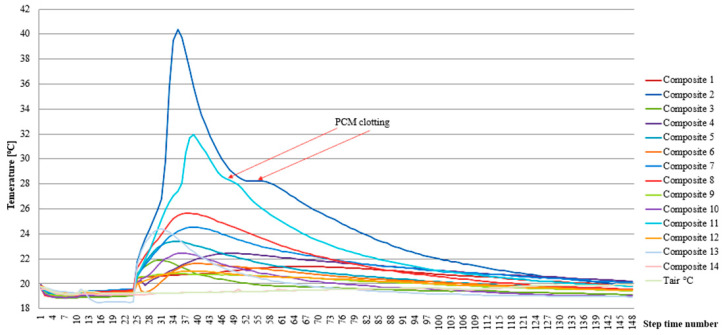
Temperature variation plot for PCM heat accumulator samples with cement matrix during matrix setting.

The exact temperature distribution over time of the cement matrix specimens is presented in Figure 13. A clear increase in the temperature of each of the samples tested was observed, which is a consequence of the exothermic heat from the hydration of the cement matrix. The magnitude of the maximum temperature rise of the individual specimens was closely related to the cement matrix content of each specimen, as evidenced by the temperature distributions of composite specimens 2 and 11. The effect of stabilising the rapid temperature changes of the samples through the PCM contained within them was also apparent, which is particularly noticeable in Figure 13 for the cooling of specimens 2 and 11.

**Figure 14:**
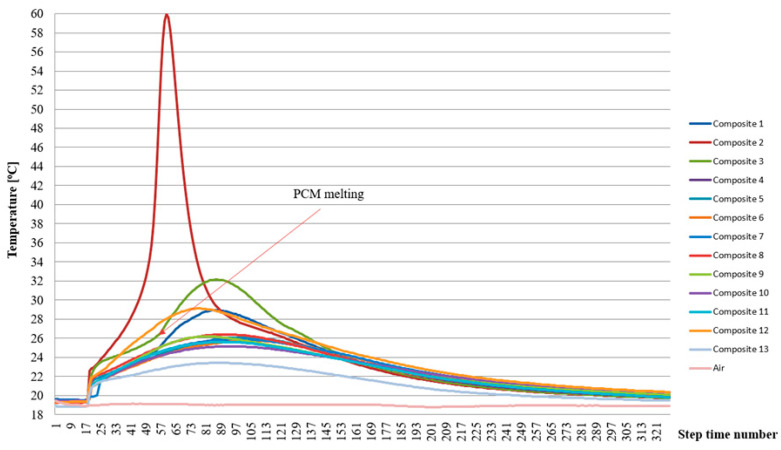
Temperature change plot for PCM heat accumulator samples with epoxy matrix during matrix setting.

Similar relationships were observed for the epoxy matrix samples, with the difference that the temperature changed more rapidly (faster heating and cooling) and reached higher maximum temperatures (up to 60 °C in the case of composite specimen 2). A clear delay in the time of pronounced temperature rises of the epoxy matrix heat accumulator samples was also observed, which is also partial to the same time indicative of the significantly lower heat capacity of the epoxy matrix relative to the cement matrix and the more exoenergic setting process of the epoxy resin compared to that of the cement. The exact temperature distribution over time of the epoxy matrix samples is presented in Figure 14.

The actual heat load tests carried out in the climate chamber proved the significant and noticeable influence of the molecular silica sieve content on the stabilisation of the temperature variation in the individual heat accumulator samples, demonstrating the effective saturation of the organic PCM RT28HC. As expected, a qualitative relationship was observed between the quantity of PCM-saturated molecular sieves in the heat accumulator sample on the ability to stabilise the temperature changes of the sieves. During the course of the initial evaluation of the results, the heating and cooling delay of each of the test samples was analysed at human thermal comfort limits, such as 20 °C and 26 °C. The content of the cement fraction causes an increase in sample temperature; however, the variable content of the carbon recyclate fraction and molecular sieves from PCM limits the temperature increase of some samples.

**Figure 15:**
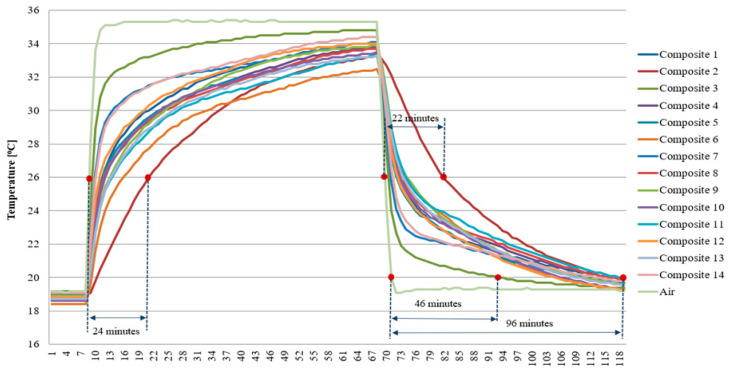
Temperature changes in the heat accumulators with cement matrix PCM molecular sieves.

A delay of 24 min in reaching the 26 °C temperature (containing the most PCM of composite sample 1) was observed relative to the change in air temperature. At the same time, during cooling down, composite sample 1 alone had the longest cooling time compared to the change in air temperature below 2 °C, which was 22 min. In contrast, the lower end of the air temperature considered comfortable (20 °C) was reached by the most efficient composite sample 1 only after 96 min and the least efficient composite sample 3 after 46 min. The exact temperature distribution over time of the cement matrix specimens is presented in Figure 15.

**Figure 16:**
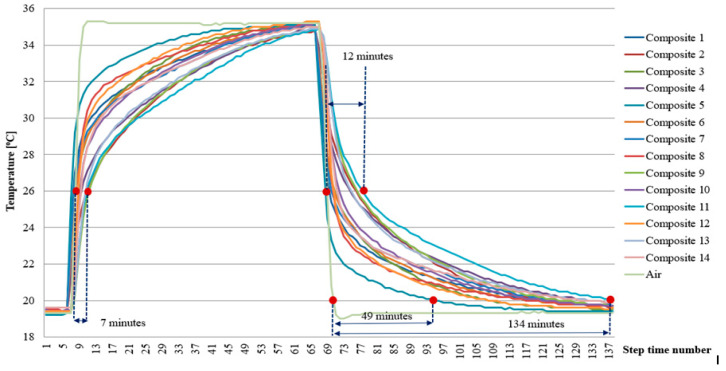
Temperature changes in the heat accumulators with epoxy matrix PCM molecular sieves.

Similar trends were observed for the epoxy matrix heat accumulator samples. As in the case of cement matrix samples, there was a qualitative relationship between the content of PCM-saturated molecular sieves in a sample on the ability to stabilise the temperature changes of the sieves. An initial evaluation of the results was carried out for identical human thermal comfort limits of 20 °C and 26 °C. In this case, the delayed heating to 26 °C (for composite sample 1 with the highest PCM content) relative to the change in air temperature was 7 min. In contrast, during cooling down, composite sample 1 alone had the longest cooling time compared to the change in air temperature below 26 °C, which was 12 min. In contrast, the lower end of the air temperature that is considered comfortable (20 °C) was reached by the most efficient composite sample 1 only after 134 min, and the least efficient composite sample 5 after 49 min. The exact temperature distribution over time of the epoxy matrix samples is presented in Figure 16.

From the results presented, it can be concluded that a higher heat storage efficiency was found in the cement matrix heat accumulator samples compared to samples of identical composition but at 26 °C for the epoxy matrix. On the other hand, at 20 °C, an increase in the cooling time of the epoxy samples relative to the cement matrix samples was found. This unequivocally demonstrated the reduced superheating time of the epoxy matrix heat accumulators in the temperature range above the indoor air thermal comfort (26 °C). At the same time, the epoxy matrix heat accumulators, compared to the cement matrix heat accumulators, had a more efficient heat distribution in the desired temperature range (20–26 °C). This fact is confirmed in Figure 13 and Figure 14 and applies to all heat accumulator samples tested.

#### 4.1.2. Results of Thermal Imaging Tests

**Figure 17:**
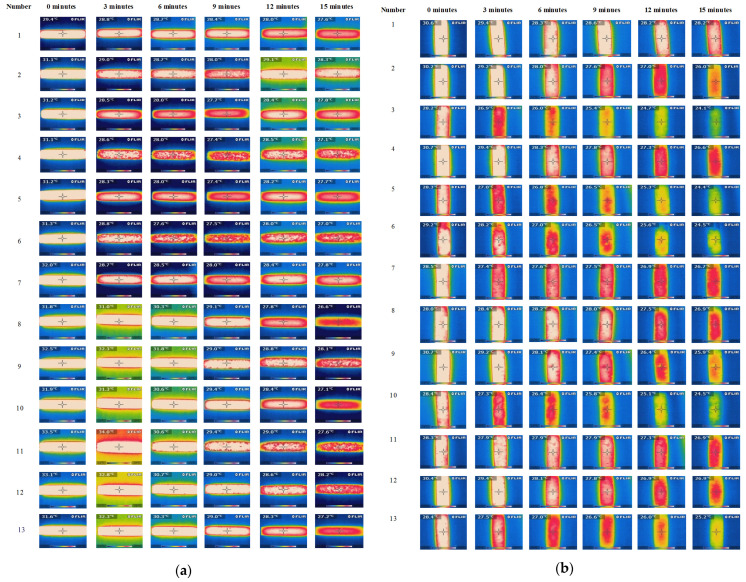
(**a**)—Compilation of thermal images of the epoxy matrix specimens during the cooling process of fully heated specimens; (**b**)—Compilation of thermal images of the cement matrix specimens during the cooling process of fully heated specimens.

The thermal images obtained, showing maps of the external surface temperature of the cement matrix and epoxy matrix heat accumulator samples, qualitatively confirmed the results of the heat load tests in the climate chamber. A summary of the thermal photographs for the epoxy matrix and cement matrix specimens is given in Figure 17.

### 4.2. Results of the Statistical Analysis

#### 4.2.1. Heat Load Tests of Cement Matrix and Epoxy Matrix Samples

The empirical results produced and separated allowed statistical analysis and the determination of an approximating function of the three dependent variables (values of volume fractions of each heat accumulator component) relative to the two output quantities (bending strength and thermal efficiency ratios of the heat accumulator samples). A summary of the empirical results from the individual experiments established in the experimental plan is shown in Table 3.

**Figure 18:**
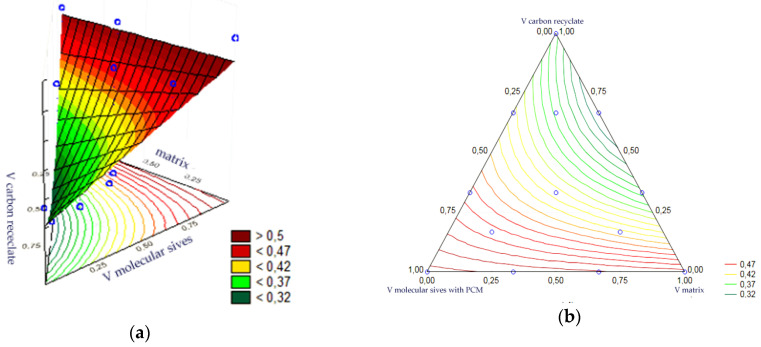
(**a**,**b**)—Charts of the obtained approximating function as a response plane.

Statistical analysis of the volume fractions and their correlations allowed us to determine their effect on the output as an indicator of the thermal efficiency of the epoxy matrix heat accumulator. Statistical significance was demonstrated for the volume fraction of PCM-saturated molecular sieves, the volume fraction of epoxy matrix and the volume fraction of carbon recyclate. The remaining correlations of the input variables were statistically insignificant and included in the error component, which amounted to MS = 0.0041. The most statistically significant input was the content of PCM-saturated molecular sieves. In turn, the charts of the resulting approximating function as a response plane are shown in Figure 18a,b. The summary of statistical results is presented in Table 4.

**Figure 19:**
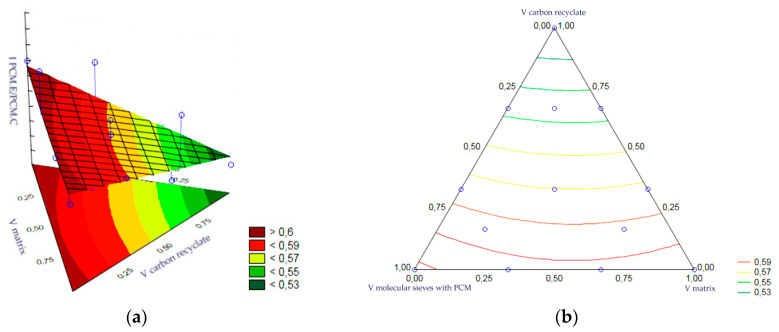
(**a**,**b**)—Charts of the obtained approximating function as a response plane.

The statistical analysis of the volume fractions and their correlations allowed us to determine their effect on the output as an indicator of the thermal efficiency of the cement matrix heat accumulator. Statistical significance was demonstrated for the volume fraction of PCM-saturated molecular sieves, the volume fraction of epoxy matrix and the volume fraction of carbon recyclate. The remaining correlations of the input variables were statistically insignificant and included in the error component, which amounted to MS = 0.0023. At the same time, the level of significance of each input was similar. In turn, the charts of the resulting approximating function as a response plane are shown in Figure 19a,b. The summary of statistical results is presented in Table 5.

#### 4.2.2. Heat Load Tests of Cement and Epoxy Matrix Samples after the Frost Resistance Tests

A summary of the empirical results obtained for the individual heat load experiments established in the experimental plan of the epoxy matrix and cement matrix heat accumulator samples is listed in Table 6.

**Figure 20:**
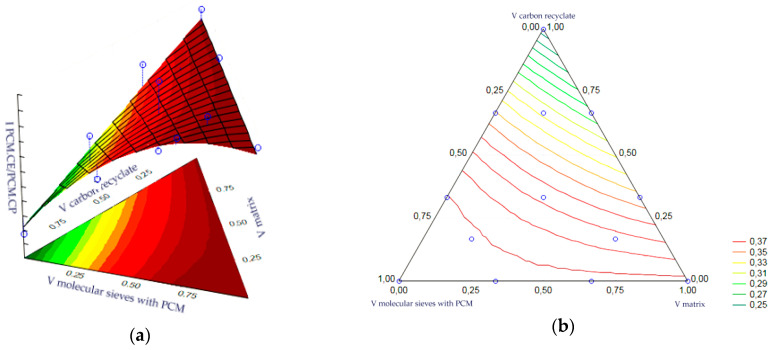
(**a**,**b**)—Charts of the obtained approximating function as a response plane.

The statistical analysis of the volume fractions and their correlations allowed us to determine their effect on the output as an indicator of the thermal efficiency of the epoxy matrix heat accumulator samples after frost resistance testing. Statistical significance was demonstrated for the volume fraction of PCM-saturated molecular sieves, the volume fraction of epoxy matrix and the volume fraction of carbon recyclate. The remaining correlations of the input variables were statistically insignificant and included in the error component, which amounted to MS = 0.0010. The level of significance was the highest for the matrix content. In turn, the charts of the resulting approximating function as a response plane are shown in Figure 20a,b. The summary of statistical results is presented in Table 7.

**Figure 21:**
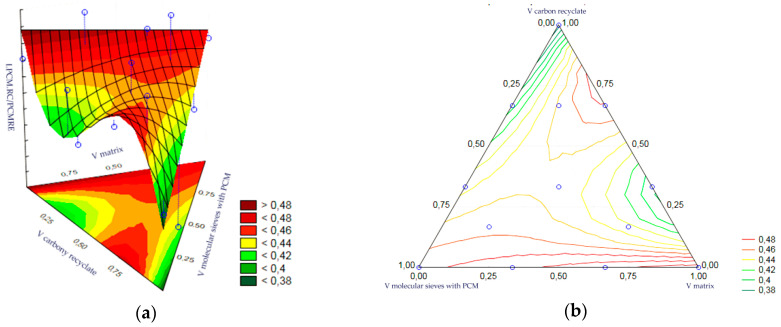
(**a**,**b**)—Charts of the obtained approximating function as a response plane.

The statistical analysis of the volume fractions and their correlations allowed us to determine their effect on the output as an indicator of the thermal efficiency of the cement matrix heat accumulator samples after frost resistance testing. Statistical significance was demonstrated for the volume fraction of PCM-saturated molecular sieves, the volume fraction of epoxy matrix and the volume fraction of carbon recyclate. The remaining correlations of the input variables were statistically insignificant and included in the error component, which amounted to MS = 0.0022. The level of significance was the highest for the matrix content. In turn, the charts of the resulting approximating function as a response plane are shown in Figure 21a,b. The summary of statistical results is presented in Table 8.

#### 4.2.3. Destructive Testing of the Cement and Epoxy Matrix Specimens

A summary of the empirical results obtained for the strength test experiments set by the experimental design of the epoxy and cement matrix heat accumulator samples is summarised in Table 9.

**Figure 22:**
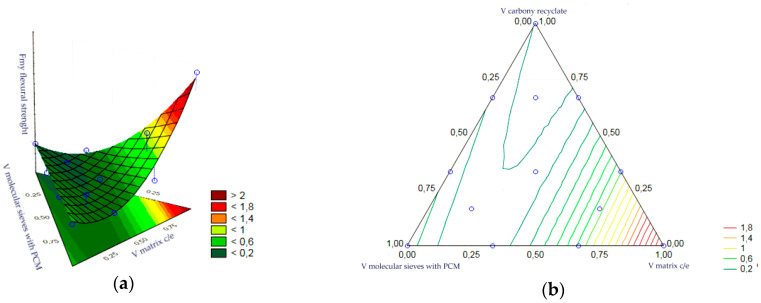
(**a,b**)—Charts of the obtained approximating function as a response plane.

The statistical analysis of volume fractions and their correlations allowed us to determine their impact on the initial value as the bending strength of heat accumulator samples with an epoxy matrix. The following are statistically significant: the volume fraction of the epoxy matrix, the volume fraction of molecular sieves, the correlation of the molecular sieve content to the matrix content and the correlation of the matrix content with carbon recyclate content. The remaining correlations of the input variables were statistically insignificant and included in the error component, which amounted to MS = 0.0384. The level of significance was the highest for the matrix content. In turn, the graphs of the obtained approximating function as the response plane are presented in Figure 22a,b. The summary of statistical results is presented in Table 10.

**Figure 23:**
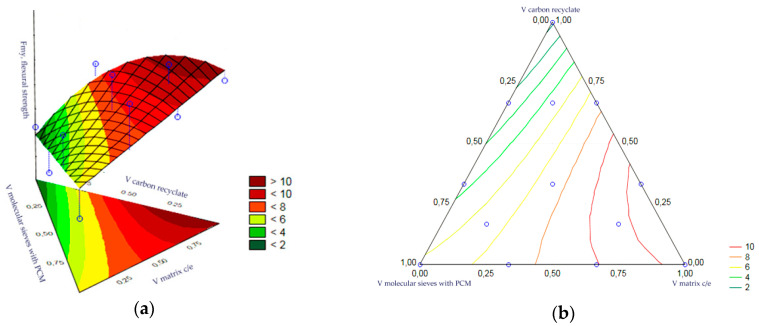
(**a**,**b**)—Charts of the obtained approximating function as a response plane.

A statistical analysis of the volume fractions and their correlations allowed us to determine their effect on the output as bending strength of the cement matrix heat accumulator samples. The statistical significance of the volume fraction of the epoxy matrix and the volume fraction of the molecular sieves was demonstrated. The remaining correlations of the input variables were statistically insignificant and included in the error component, which amounted to MS = 0.0541. The level of significance was the highest for the matrix content. In turn, the charts of the resulting approximating function as a response plane are shown in Figure 23a,b. The summary of statistical results is presented in Table 11.

#### 4.2.4. Destructive Tests of Cement and Epoxy Matrix Samples after the Frost Resistance Tests

A summary of the empirical results obtained for the strength test experiments established in the experimental plan of the epoxy and cement matrix heat accumulator samples after frost resistance testing is summarised in Table 12.

**Figure 24:**
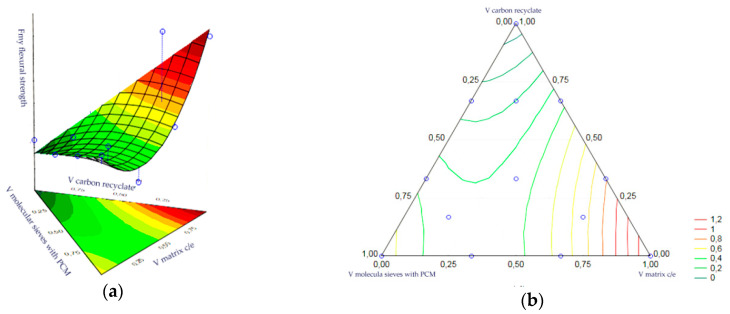
(**a**,**b**)—Charts of the obtained approximating function as a response plane.

The statistical analysis of the volume fractions and their correlations allowed us to determine their effect on the output as the bending strength of the epoxy matrix heat accumulator samples after frost resistance testing. Statistical significance was demonstrated for the volume fraction of the epoxy matrix and the volume fraction of the molecular sieves. The remaining correlations of the input variables were statistically insignificant and included in the error component, which amounted to MS = 0.0502. The level of significance was the highest for the matrix content. In turn, the charts of the resulting approximating function as a response plane are shown in Figure 24a,b. The summary of statistical results is presented in Table 13.

**Figure 25:**
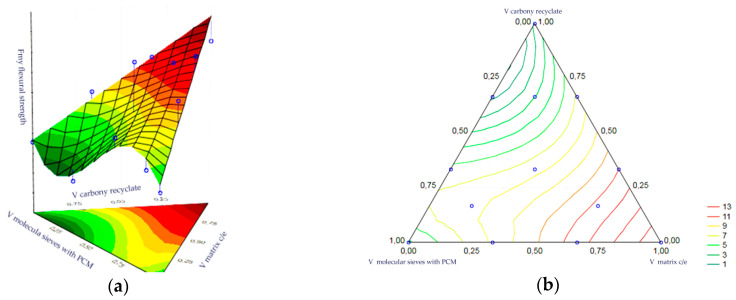
(**a**,**b**)—Charts of the obtained approximating function as a response plane.

The statistical analysis of the volume fractions and their correlations allowed us to determine their effect on the output as the bending strength of the cement matrix heat accumulator samples after frost resistance testing. Statistical significance was demonstrated for the volume fraction of the epoxy matrix and the volume fraction of the molecular sieves. The remaining correlations of the input variables were statistically insignificant and included in the error component, which amounted to MS = 0.0535. The level of significance was the highest for the matrix content. In turn, the charts of the resulting approximating function as a response plane are shown in Figure 25a,b. The summary of statistical results is presented in Table 14.

The resulting approximating polynomials showing the relationship between the content of the individual components of the PCM heat accumulators and their heating, cooling, heat distribution and bending/bending strength efficiencies, both before and after being subjected to cyclic freezing and thawing, are summarised in Table 15.

**Figure 26:**
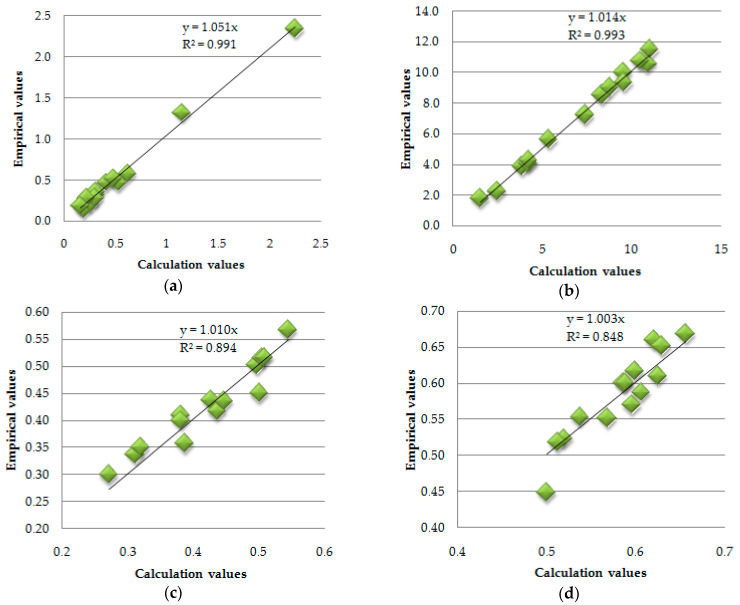

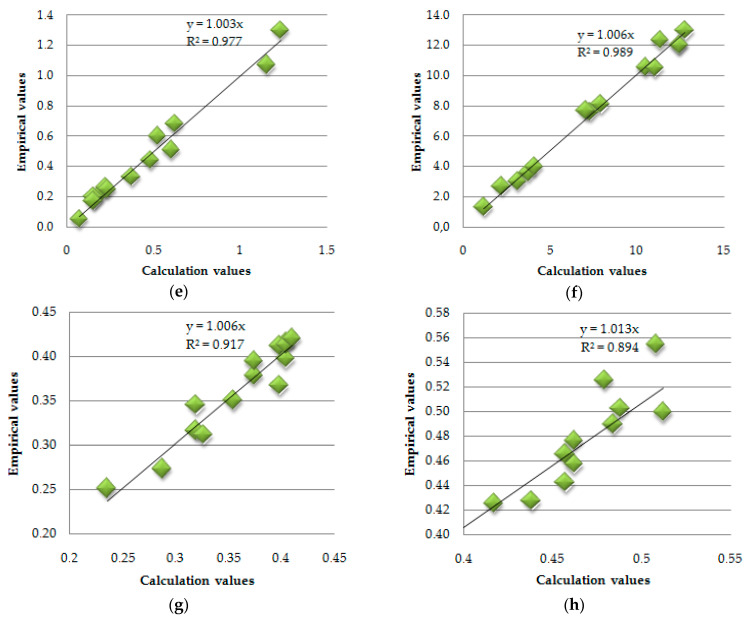
Summary scatter graphs for the empirical input quantities and those calculated from the produced response functions, (**a**)-graph for cement samples, not frozen, for flexural strength values; (**b**)-graph for cement samples, not frozen, for Heat load values; (**c**)-graph for cement samples after freezing for flexural strength values; (**d**)-graph for cement samples after freezing for Heat load values; (**e**)-graph for epoxy samples, not frozen, for flexural strength values; (**f**)-graph for epoxy samples, not frozen, for Heat load values; (**g**)-graph for epoxy samples after freezing for flexural strength values; (**h**)-graph for epoxy samples after freezing for Heat load values.

Given the sensitivity of the approximating polynomial functions to the peak values of the results, decisions were made to additionally verify the obtained functions by analysing the variance of the computed results with specifically separated empirical results. Calculations of the Snedecor–Fischer test and an analysis of the statistical test result by the quasi-Newton method were performed according to [13], and the procedure was expressed in Equations (6)–(9). In each of the cases considered, the value of the variance quotient was lower than the value of the critical variance. This proved the condition of the statistical test. An example of the results of a scatter analysis of calculated versus empirical quantities is shown in Figure 26. The summary of the statistical test results is presented in Table 16.

The values shown in Table 16 for the comparison of the variances of the empirical and theoretical quantities with respect to the critical quantities are close to and smaller than the critical quantities, which confirms that the null hypothesis condition was met for each of the eight types of experiments analysed.

Moreover, the scatter charts of empirical and theoretical values presented in Figure 26, as well as the values of the directional coefficients ‘a’ and the coefficients of determination ‘R^2^’, which are close to unity, prove there is an acceptable level of error of the produced approximating polynomial functions and the adequacy of the adopted calculation model.

## 5. Discussion and Conclusions

### 5.1. Vacuum Saturation of Molecular Silica Sieves with Liquid Organic PCM

The completed research enabled a multi-faceted analysis of the physical and thermal characteristics of new composite heat accumulators containing pressure PCM-saturated molecular silica sieves.

**Figure 27:**
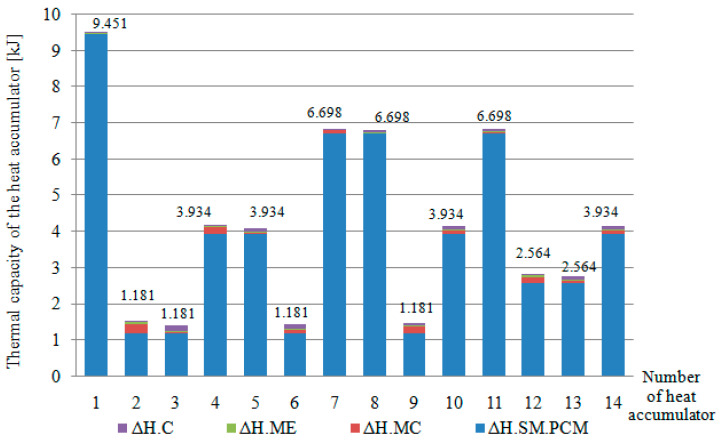
Summary of the heat capacity of all heat accumulators and their components tested. ΔH.C—heat capacity of the carbon recyclate; ΔH.ME—heat capacity of the epoxy matrix; ΔH.MC—heat capacity of the cement matrix; ΔH.SM.PCM—heat capacity of the PCM-saturated molecular sieves.

Below is a summary of the most important observations and discussion of the results in each of the considered aspects of the functioning of the new PCM composites. One of the main results of the research was the development of a simple and effective method for the pressurised saturation of granular molecular silica sieves with liquid organic PCM. Providing a vacuum of 85–100 kPa allowed the saturation of molecular sieves with pore sizes of 4–5 A to be approximately 70%. The leak-tightness of the PCM-saturated molecular sieves was assessed as satisfactory since, after the PCM-saturated molecular sieves cooled below 23 °C and cleaned of any residual uninfiltrated PCM, a slight leakage of small amounts of PCM was found during cyclic heating. The leakage was only observed for the first 8–10 heating cycles. This indicates that only the PCM contained in the outer layer of the molecular sieve was leaking, while the rest of the PCM (the vast majority), due to the small pore size of 4–5 A and the adhesion and cohesion forces of the liquid PCM, remained embedded in the molecular sieve structure. The choice of molecular sieve as the medium for PCM application largely negated the problem of PCM leakage during melting while allowing the significant heat capacity of the 256 cm^3^ composite heat accumulators to be achieved. The heat capacity values of the heat accumulators tested are shown in Figure 27.

**Figure 28:**
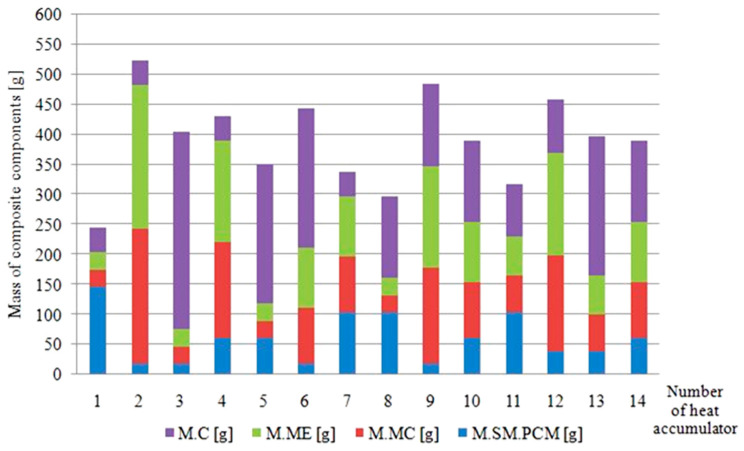
Summary of the masses of the heat accumulator tested and their components. M.C—mass of the carbon recyclate; M.ME—mass of the epoxy matrix; M.MC—mass of the cement matrix; M.SM.PCM—mass of the PCM-saturated molecular sieves.

In the further part of the discussion, the results regarding the PCM molecular sieve impregnation method, the mechanical strength of the composites, the obtained mathematical models of the approximating functions, the properties of the matrices used and the thermal properties of the composites were discussed in detail. The high density of heat and cold storage achieved through the use of the enthalpy of melting/solidification of the PCM made it possible to significantly reduce the weight of the heat accumulators, which is an undoubted advantage of the heat accumulators studied in the context of their use as passive heat and cold storage to stabilise indoor climate conditions in the cabins of electric vehicles or in the ventilation systems of buildings. A summary of the masses of the individual components of the heat accumulators tested is shown in Figure 28.

In a summary of the data in Figure 27 and Figure 28, sample 1 containing the highest concentration of PCM-saturated molecular sieves (80%) achieved a heat capacity of 9.451 kJ at a volume of 256 cm^3^ and a total mass of 167 g.

### 5.2. Effect of the Matrix Setting Heat on the Durability and Characteristics of the Heat Accumulator

An important aspect of the analysis was the choice of composite matrix in the form of a cement mixture and epoxy resin. This is important both in terms of the water-binding affinity of its structure and the exoenergetic binding of the cement and epoxy resin. Excessive heating of the composite mix prior to the mix setting will cause melting and convective release of the PCM from the molecular sieves.

Finally, the PCM silica sieves within the matrix were fully exuded. Anomalies in the form of unsealed molecular sieves located in the immediate flank of the heat accumulator reduced their local strength and were a source of PCM leakage.

In addition, the sensitivity of molecular sieves to prolonged exposure to water and their cyclic freezing and thawing in aqueous or high-humidity environments has been observed. A similar phenomenon was found during cement matrix setting, where the high hydration heat of the cement and the aqueous environment caused increased the destruction of the molecular sieves, thus releasing the PCM contained in them. This phenomenon simultaneously influenced the formation of micro-cracks throughout the heat accumulator, reducing its strength, frost resistance and leak-tightness capabilities. A correlation of the frequency of these adverse phenomena with increasing contents of the cement matrix and PCM molecular sieves quantities was observed.

### 5.3. Heat Distribution Efficiency Characteristics of the Tested PCM Heat Accumulator Types

The experimentally verified efficiency of heat distribution directly depends on the content of the PCM-saturated molecular sieves, as demonstrated in Figure 13, Figure 14, Figure 15 and Figure 16 and during qualitative examinations that used a thermal imaging camera (Table 3 and Table 4). The heat storage capacity was unambiguously observed by delaying the heat accumulator’s arrival to a temperature of 26 °C by 24 min for the cement matrix and 7 min for the epoxy matrix, relative to the outside air temperature. At the same time, it was found in the heat accumulators with the highest heat capacity that the cooling of the samples from 35 °C to 26 °C was delayed by 22 min for the cement matrix samples and 12 min for the epoxy matrix samples, respectively, compared to the changing ambient air temperature. This demonstrates that, when combined in the same proportions, the epoxy matrix and carbon recyclate matrix, compared to the cement matrix and carbon recyclate matrix, allows the heat accumulator to charge faster and, in the case of overheating, to cool down to 26 °C. At the same time, cooling of the epoxy matrix samples compared to the cement matrix samples in the temperature range of 26–20 °C took longer. This demonstrates a more effective distribution of stored heat and an increase in the effective thermal capacity of the heat accumulator as an increase in the share of PCM undergoing the phase change. These differences were found in all the heat accumulators tested but with varying intensities. The increase in heat storage time was in the range of 38–3 min, an improvement of 28.36% to 6.51%. This relationship was further confirmed by qualitative thermal imaging examinations shown in Figure 17.

### 5.4. Bending Strength and Frost Resistance of PCM Heat Accumulators

For both cement matrix and epoxy matrix heat accumulators, the decisive parameter for bending strength was the content of the matrix itself and, secondarily, the content of PCM-saturated molecular sieves as a factor in reducing the strength properties of the individual samples. As expected, they achieved higher bending strength values at equal contents of the other components like in the cement matrix specimens. In this context, the bending strength requirements for the cement matrix cast were only met by sample 2 with 80% matrix content, while the other samples did not meet the requirements. At the same time, the cement matrix samples were clearly more sensitive to loss of strength under cyclic freezing and thawing. The recorded bending strength reduction after frost resistance testing was within 2–49%, while the epoxy matrix samples suffered bending strength reduction by 3–23.2%.The bending strength values of the individual epoxy matrix specimens were within 10.48–1.54 MPa, which, together with the heat load resistance, the acceptable leak-tightness of the PCM and the satisfactory thermal and physical properties after frost resistance testing allows for use both as composite heat-accumulating window sills and as heat accumulators for electric vehicles.

### 5.5. Statistical Significance of Individual Components on Heat Storage Efficiency and Durability of Heat Accumulators

The obtained empirical results and the results of the statistical analysis allowed us to determine which input quantities (volume fractions of molecular silica sieves vacuum saturated with organic PCM RT28, carbon recyclate and cement and epoxy matrices) contributed to the heat transfer efficiency of the composite heat accumulator and its bending strength. Consequently, all three input quantities considered were statistically significant for heat transfer efficiency, regardless of whether the heat accumulators had a cement or epoxy matrix and whether or not the samples were subjected to cyclic freezing and thawing. The parameter most statistically significant to the efficiency of heat distribution for the cement matrix specimens was the molecular sieve content, with all three input quantities having similar levels of significance for the epoxy matrix. In contrast, the heat accumulator samples subjected to cyclic freezing and thawing were characterised by approximately 10–22% lower heat distribution efficiency values, while the statistically significant parameters of cement matrix samples remained unchanged. In the case of epoxy matrix samples, the statistical significance of the carbon recyclate content was reduced. Regarding the influence of the values of the individual components of the composite heat accumulators on the bending strength of the entire composite, statistically significant to the cement matrix specimens are the matrix content, followed by (and at a significance level three to four times lower) the correlations of the input quantities and the content of the molecular sieves. In contrast, for the epoxy matrix heat accumulators, the matrix content and, secondarily, the molecular sieve content were statistically significant. In contrast, the matrix content, followed by (and at a significance level two to four times lower) the PCM-saturated molecular sieve content, was statistically significant to the strength of the heat accumulator samples with both matrix types tested for frost resistance.

### 5.6. Range of Applicability of the Resulting Equations

The obtained approximation functions of the volume fractions of the individual three components of the heat accumulators and their correlations made it possible to predict the heat storage efficiency and the bending strength of future heat accumulators containing the untested ratios of the three components of interest. It proved which input quantities are statistically significant at the assumed level of error. In addition, the functions met the conditions of the null hypothesis of the Snedecor–Fischer statistical test and the scatter plots of the theoretical and empirical quantities, while the values of the directional and determination coefficients obtained by quasi-Newtonian analysis confirmed the error rate and the adequacy of the approximating functions obtained.

This multi-faceted analysis of PCM (phase-change material) heat accumulators, in terms of thermophysics, durability and technology, was completed to justify their large-scale use in both construction and electrical vehicles. We see a need for further research into improving the heat storage capacity of composite heat accumulators formed based on the basis of phase-change materials through the application of non-congruent hydrated salts, light metals and nano-liquid emulsions, which can help, on the one hand, to increase the thermal capacity of the PCM while improving its solid-state thermal conductivity. Proper use of nanofluids to create new PCM composites may enable a level of heat distribution between the PCM and the external environment to be achieved, which was previously unrecorded.

## 6. Conclusions

The most important results and observations obtained as part of this research are summarized, and the following conclusions are formulated:

- Silica molecular sieves are a good porous material for soaking under pressure with organic phase-change material from the group of saturated aliphatic hydrocarbons. The most preferred matrix for composites containing carbon recyclate and silica molecular sieves with PCM is an epoxy matrix.
- The advantage of the epoxy matrix over the cement matrix is visible in the aspects of bending strength, frost resistance, frost resistance and tightness.
- The epoxy matrix has a much less destructive effect on the silica molecular sieves during its binding process.
- The obtained response functions are a statistically verified and useful tool for predicting the thermophysical properties of heat accumulators based on silica molecular sieves, carbon recyclate and cement and epoxy matrices.

The paper is consistent with the subject of efficient distribution of heat from renewable sources, recycling, circular economy, autonomous and low-carbon construction, innovative building materials and sustainable construction.

## Figures and Tables

**Table 1:** Summary of the ranges of variation for the input quantities of the experimental plan.

No	Input Value	Minimum Value	Maximum Value
1	Molecular sieves with PCM	0.1	0.8
2	Carbon recyclate	0.1	0.8
3	Concrete matrix/epoxy matrix	0.1	0.8

**Table 2:** Summary of input and output quantities for the experiments carried out.

No	Input Values		
	Volume Fraction of Molecular Sieves from PCM	Volume Fraction of Cement or Epoxy Matrix	Volume Fraction of Carbon Recyclate
symbol	V_SM.PCM_	V_MC.ME_	V_C_
unit	[−]	[−]	[−]
1	0.800	0.100	0.100
2	0.100	0.800	0.100
3	0.100	0.100	0.800
4	0.333	0.567	0.100
5	0.333	0.100	0.567
6	0.100	0.333	0.567
7	0.567	0.333	0.100
8	0.567	0.100	0.333
9	0.100	0.567	0.333
10	0.333	0.333	0.333
11	0.567	0.217	0.217
12	0.217	0.567	0.217
13	0.217	0.217	0.567
14	0.333	0.333	0.333

**Table 3:** Summary of input and output quantities for the completed experiments.

No	Input Values			Output Values	
	Volume Fraction of Molecular Sieves from PCM	Volume Fraction of Matrix	Volume Fraction of Carbon Recyclate	Thermal Efficiency Index of Cement Matrix Samples	Thermal Efficiency Index of Epoxy Matrix Samples
symbol	V_SM.PCM_	V_MC.ME_	V_C_	I_T.MC_	I_T.ME_
unit	[−]	[−]	[−]	[−]	[−]
1	0.800	0.100	0.100	0.544	0.620
2	0.100	0.800	0.100	0.496	0.596
3	0.100	0.100	0.800	0.386	0.519
4	0.333	0.567	0.100	0.504	0.599
5	0.333	0.100	0.567	0.380	0.537
6	0.100	0.333	0.567	0.271	0.625
7	0.567	0.333	0.100	0.426	0.656
8	0.567	0.100	0.333	0.508	0.629
9	0.100	0.567	0.333	0.436	0.586
10	0.333	0.333	0.333	0.311	0.606
11	0.567	0.217	0.217	0.446	0.500
12	0.217	0.567	0.217	0.380	0.568
13	0.217	0.217	0.567	0.319	0.512
14	0.333	0.333	0.333	0.500	0.588

**Table 4:** Summary of statistical results produced for the epoxy matrix heat accumulator samples.

Input Quantities	Simplex–Centroid Design of the Experiment. R^2^ = 0.8806; Plan for Three-Constrained Mixtures					
	Factor	Standard Error	t(10)	*p*	−95% Gran.	+95% Gran.
(A) V molecular sieves with PCM	0.503768	0.042141	11.95430	0.000000	0.409872	0.409872
(B) V matrix	0.484694	0.050237	9.64817	0.000002	0.372759	0.372759
(C) carbon recyclate	0.353824	0.050237	7.04312	0.000035	0.241889	0.241889
BC	−0.389456	0.252705	−1.54115	0.154306	−0.952519	−0.952519

**Table 5:** Summary of statistical results produced for the cement matrix heat accumulator samples.

Input Quantities	Simplex–Centroid Design of the Experiment. R^2^ = 0.9056; Plan for Three-Constrained Mixtures					
	Factor	Standard Error	t(10)	*p*	−95% Gran.	+95% Gran.
(A) V molecular sieves with PCM	0.630949	0.071899	8.77545	0.000005	0.470747	0.791151
(B) V matrix	0.624738	0.071899	8.68906	0.000006	0.464536	0.784940
(C) carbon recyclate	0.505985	0.044748	11.30741	0.000001	0.406280	0.605690
AB	−0.064498	0.379402	−0.17000	0.868401	−0.909857	0.780862

**Table 6:** Summary of input and output quantities for the experiments carried out after the frost resistance tests.

No	Input Values			Output Values	
	Volume Fraction of Molecular Sieves from PCM	Volume Fraction of Matrix	Volume Fraction of Carbon Recyclate	Thermal Efficiency Index of Cement Matrix Samples	Thermal Efficiency Index of Epoxy Matrix Samples
symbol	V_SM.PCM_	V_MC.ME_	V_C_	I_T.MC_	I_T.ME_
unit	[−]	[−]	[−]	[−]	[−]
1	0.800	0.100	0.100	0.404	0.457
2	0.100	0.800	0.100	0.404	0.462
3	0.100	0.100	0.800	0.235	0.394
4	0.333	0.567	0.100	0.398	0.508
5	0.333	0.100	0.567	0.319	0.323
6	0.100	0.333	0.567	0.287	0.457
7	0.567	0.333	0.100	0.374	0.479
8	0.567	0.100	0.333	0.398	0.417
9	0.100	0.567	0.333	0.374	0.462
10	0.333	0.333	0.333	0.319	0.438
11	0.567	0.217	0.217	0.404	0.512
12	0.217	0.567	0.217	0.326	0.370
13	0.217	0.217	0.567	0.354	0.488
14	0.333	0.333	0.333	0.410	0.484

**Table 7:** Summary of statistical results produced for the epoxy matrix heat accumulator samples after frost resistance testing.

Input Quantities	Simplex–Centroid Design of the Experiment, R^2^ = 0.9237; Plan for Three-Constrained Mixtures					
	Factor	Standard Error	t(10)	*p*	−95% Gran.	+95% Gran.
(A) V molecular sieves with PCM	0.393833	0.024949	15.78535	0.000000	0.338243	0.449423
(B) V matrix	0.392878	0.020929	18.77221	0.000000	0.346246	0.439510
(C) carbon recyclate	0.244442	0.024949	9.79755	0.000002	0.188851	0.300032
AC	−0.211588	0.125502	1.68594	0.122706	−0.068048	0.491223

**Table 8:** Summary of statistical results produced for the cement matrix heat accumulator samples after frost resistance testing.

Input Quantities	Simplex–Centroid Design of the Experiment. R^2^ = 0.9337; Plan for Three-Constrained Mixtures					
	Factor	Standard Error	t(10)	*p*	−95% Gran.	+95% Gran.
(A) V molecular sieves with PCM	0.467022	0.030853	15.13697	0.000000	0.39828	0.535767
(B) V matrix	0.496569	0.032728	15.17267	0.000000	0.42365	0.569491
(C) carbon recyclate	0.375909	0.032728	11.48590	0.000000	0.30299	0.448831
BC (B-C)	−0.729940	0.398828	−1.83021	0.097142	−1.61858	0.158704

**Table 9:** Summary of input and output quantities for the experiments carried out before the frost resistance tests.

No	Input Values			Output Values	
	Volume Fraction of Molecular Sieves from PCM	Volume Fraction of Matrix	Volume Fraction of Carbon Recyclate	Flexural Strength of Cement Matrix Samples	Flexural Strength of Epoxy Matrix Samples
symbol	V_SM.PCM_	V_MC.ME_	V_C_	F_MC_	F_ME_
unit	[-]	[-]	[-]	[MPa]	[MPa]
1	0.800	0.100	0.100	0.41	3.86
2	0.100	0.800	0.100	2.25	9.53
3	0.100	0.100	0.800	0.20	2.49
4	0.333	0.567	0.100	0.53	8.32
5	0.333	0.100	0.567	0.19	1.54
6	0.100	0.333	0.567	0.31	8.78
7	0.567	0.333	0.100	0.26	10.89
8	0.567	0.100	0.333	0.31	7.40
9	0.100	0.567	0.333	0.62	9.57
10	0.333	0.333	0.333	0.30	4.22
11	0.567	0.217	0.217	0.48	5.37
12	0.217	0.567	0.217	1.15	11.02
13	0.217	0.217	0.567	0.22	4.25
14	0.333	0.333	0.333	0.16	10.48
15	0	100	0	6.34	30.45

**Table 10:** Summary of bending strength statistical results produced for the epoxy matrix heat accumulator samples.

Input Quantities	Simplex–Centroid Design of the Experiment. R^2^ = 0.9106; Plan for Three-Constrained Mixtures					
	Factor	Standard Error	t(10)	*p*	−95% Gran.	+95% Gran.
(A) V molecular sieves with PCM	0.46507	0.153871	3.02246	0.014422	0.11699	0.81315
(B) V matrix	2.15395	0.178889	12.04073	0.000001	1.74928	2.55862
(C) V carbon recyclate	0.19459	0.153871	1.26460	0.237778	−0.15349	0.54267
AB	−3.51328	0.772809	−4.54611	0.001394	−5.26149	−1.76506
BC	−2.74871	0.772809	−3.55678	0.006150	−4.49693	−1.00050

**Table 11:** Summary of bending strength statistical results produced for the cement matrix heat accumulator samples.

Input Quantities	Simplex–Centroid Design of the Experiment, R^2^ = 0.9061; Plan for Three-Constrained Mixtures					
	Factor	Standard Error	t(10)	*p*	−95% Gran.	+95% Gran.
(A) V molecular sieves with PCM	6.17547	1.529429	4.037760	0.002370	2.76769	9.58324
(B) V matrix	10.35547	1.823245	5.679695	0.000204	6.29303	14.41792
(C) carbon recyclate	1.81374	1.823245	0.994784	0.343302	−2.2471	5.87618
BC	13.21256	9.171430	1.440622	0.180258	−7.22266	33.64778

**Table 12:** Summary of input and output quantities for the experiments carried out after the frost resistance tests.

No	Input Values			Output Values	
	Volume Fraction of Molecular Sieves from PCM	Volume Fraction of Molecular Sieves from PCM	Volume Fraction of Molecular Sieves from PCM	Flexural Strength of Cement Matrix Samples	Flexural Strength of Epoxy Matrix Samples
symbol	V_SM.PCM_	V_MC.ME_	V_C_	F_MC_	F_ME_
unit	[−]	[−]	[−]	[MPa]	[MPa]
1	0.800	0.100	0.100	0.60	3.75
2	0.100	0.800	0.100	1.15	12.41
3	0.100	0.100	0.800	0.07	3.13
4	0.333	0.567	0.100	0.52	12.75
5	0.333	0.100	0.567	0.16	1.21
6	0.100	0.333	0.567	0.20	7.91
7	0.567	0.333	0.100	0.15	10.49
8	0.567	0.100	0.333	0.37	7.29
9	0.100	0.567	0.333	0.62	11.03
10	0.333	0.333	0.333	0.22	7.07
11	0.567	0.217	0.217	0.48	4.11
12	0.217	0.567	0.217	1.23	11.34
13	0.217	0.217	0.567	0.23	2.22
14	0.333	0.333	0.333	0.15	12.43
15	0	100	0	6.04	25.91

**Table 13:** Summary of bending strength statistical results produced for the epoxy matrix heat accumulator samples after frost resistance testing.

Input Quantities	Simplex–Centroid Design of the Experiment, R^2^ = 0.9271; Plan for Three-Constrained Mixtures					
	Factor	Standard Error	t(10)	*p*	−95% Gran.	+95% Gran.
(A) V molecular sieves with PCM	0.56932	0.175140	3.25066	0.008711	0.17909	0.959560
(B) V matrix	1.21019	0.175140	6.90984	0.000041	0.81995	1.600429
(C) carbon recyclate	−0.05746	0.146917	−0.39113	0.703903	−0.38481	0.269886
AB	−2.05771	0.881005	−2.33564	0.041648	−4.02071	−0.094710

**Table 14:** Summary of bending strength statistical results produced for the cement matrix heat accumulator samples after frost resistance testing.

Input Quantities	Simplex–Centroid Design of the Experiment. R^2^ = 0.8932; Plan for Three-Constrained Mixtures					
	Factor	Standard Error	t(10)	*p*	−95% Gran.	+95% Gran.
(A) V molecular sieves with PCM	4.83237	1.61602	2.990301	0.013567	1.2317	8.43308
(B) V matrix	14.85199	1.52344	9.748968	0.000002	11.4575	18.24643
(C) carbon recyclate	3.27421	1.61602	2.026101	0.070261	−0.3265	6.87492
AC(A-C)	27.62123	19.69305	1.402587	0.191014	−16.2576	71.50008

**Table 15:** Summary of the response functions produced for all completed experiments.

Function			Formula
Cement samples	Before freezing	Flexural strength	F_MC_ = 0.465·V_SM.PCM_ + 2.154·V_MC.ME_ + 0.195·V_C_ − 3.513 V_SM.PCM_·V_MC.ME_ − 2.749·V_MC.ME_·V_C_
		Thermal efficiency index	I_T.MC_ = 0.504·V_SM.PCM_ + 0.485·V_MC.ME_ + 0.354·V_C_ − 0.389 V_MC.ME_·V_C_
	After freezing	Flexural strength	F_MC_ = 0.569·V_SM.PCM_ + 1.210·V_MC.ME_ − 0.057·V_C_ − 2.05·V_SM.PCM_·V_MC.ME_
		Thermal efficiency index	I_T.MC_ = 0.394·V_SM.PCM_ + 0.393·V_MC.ME_ + 0.244·V_C_ − 0.216 V_SM.PCM_·V_C_
Epoxy samples	Before freezing	Flexural strength	F_ME_ = 6.175·V_SM.PCM_ + 10.355·V_MC.ME_ + 1.814·V_C_ + 13.213·V_MC.ME_·V_C_
		Thermal efficiency index	I_T.ME_ = 0.613·V_SM.PCM_ + 0.608·V_MC.ME_ + 0.530·V_C_ − 0.316 V_MC.ME_·V_SM.PCM_
	After freezing	Flexural strength	F_ME_ = 4.832·V_SM.PCM_ + 14.852·V_MC.ME_ + 3.274·V_C_ + 27.621 V_SM.PCM_·V_C_·(V_SM.PCM_ − V_C_)
		Thermal efficiency index	I_T.ME_ = 0.467·V_SM.PCM_ + 0.497·V_MC.ME_ + 0.376·V_C_ − 0.730 V_MC.ME_·V_C_·(V_MC.ME_ − V_C_)

**Table 16:** Summary of calculated values and critical Snedecor–Fischer statistics.

Research Case			Quotient of Variances F_PCM(α.f1.f2)_	Critical Value F_kr_
Cement samples	Before freezing	Flexural strength	2.4321	2.4800
		Heat load	2.4730	2.4800
	After freezing	Flexural strength	2.4605	2.4800
		Heat load	2.4737	2.4800
Epoxy samples	Before freezing	Flexural strength	2.4538	2.4800
		Heat load	2.4612	2.4800
	After freezing	Flexural strength	2.4775	2.4800
		Heat load	2.4297	2.4800

## Data Availability

The raw data supporting the conclusions of this article will be made available by the authors on request.

## List of Designations

**Symbol**	**Name**	**Unit**
ɛ	Emissivity	[-]
$\sigma_{a}^{2}$	Variance of inaccuracy of actual measurements	[-]
σ^2^	Variance of inaccuracies of calculation quantities	[-]
df	Degrees of freedom	[-]
F	Statistic value	[-]
f_1_, f_2_	Degrees of freedom	[-]
f_m,yk_	Characteristic bending strength	[N/mm^2^]
I_H_	Thermal efficiency heat composites	[-]
MS	Pure mistake	[-]
p	Significance level	[-]
R^2^	Coefficient of determination	[-]
SS	Variance	[-]
T_emp.ΔH PCM_	PCM phase transition temperature	[°C]
V_C_	Volume fraction of carbon recyclate	[-]
V_MC.ME_	Volume fraction of cement or resin matrix	[-]
V_SM.PCM_	Volume fraction of molecular sieves from PCM	[-]
I_T.MC_	Thermal efficiency index of cement matrix samples	[-]
I_T.ME_	Thermal efficiency index of samples with an epoxy matrix	[-]
PCM	Phase-change materials	[-]
GHE	Ground heat exchanger	[-]
LCA	Life cycle assessment	[-]
